# Determinant of Implanon discontinuation among women in southwest Ethiopia: unmatched case control study

**DOI:** 10.1186/s40834-023-00253-9

**Published:** 2023-11-03

**Authors:** Samuel Ejeta Chibsa, Kenbon Bayisa, Mustefa Adem Hussen, Bilisumamulifna Tefera Kefeni

**Affiliations:** 1 Department of Midwifery, College of Health Sciences, Mattu University, Mettu, Ethiopia; 2 Department of Public Health, College of Health Sciences, Mattu University, Mettu, Ethiopia

**Keywords:** Implanon discontinuation, Unmatched case control, South West, Ethiopia

## Abstract

**Background:**

Over 4.5 million women worldwide have used Implanon. It plays an important role in reducing unwanted conceptions, lowering maternal mortality, and enhancing child survival. As a result, the availability of family planning programmes encourages women to begin using contraception and encourages women who are already using family planning to continue using it. The purpose of this study was to investigate the factors that lead to implanon cessation among women in southwest, Ethiopia.

**Methods:**

A facility-based unmatched case–control study was conducted from February 01 to March 02, 2023. It included 348 participants, 174 cases, and 174 controls. The cases were selected consecutively, and the controls were selected using a systematic random sampling method. Data was collected through a structured, face-to-face interview and entered into Epi-data version 4.6 and SPSS version 25.0 for analysis. The confidence interval (CI) of 95 and the strength of the association were measured using an adjusted odds ratio. A *p*-value of less than 0.05 was considered statistically significant.

**Result:**

Women whose husbands have formal education [AOR = 0.33, 95% CI (0.121–0.0944)], women who have been counseled individually [AOR = 3.403 (1.390–8.3.32)], women who have been counseled for less than 5 min [AOR = 3.143, 95% CI (1.303–8.046)], and women who discuss Implanon insertion with their partner [AOR = 0.289, 95% CI (0.143–0.585)] were significantly associated with Implanon discontinuation.

**Conclusion:**

Implanon discontinuation was predicted by the husband's education, the number of women counselled alone, the length of counselling, a conversation with the spouse, satisfaction with the service, and implanon side effects. The health care provider should increase counselling services, especially the length of implanon pregnancy, in accordance with the national family planning recommendations, to reduce early implanon removal.

## Introduction

Implanon is a single-rod; etonogestrel-containing implant that, with a failure rate of less than 1%, stops unplanned pregnancies for up to three years and immediately restores fertility if it is removed [1–4]. It's primary method of action is ovulation suppression, which increases cervical mucus viscosity, which prevents spermatozoa from passing through and changes the endometrial lining [5, 6]. Although the insertion and removal of implanon need only simple surgical procedures, clients should get proper counseling regarding the methods' efficacy, length of action, potential side effects, and their right to stop using implanon [7]. The discontinuation of the most effective forms of contraception, like Implanon, despite their safety and efficacy, has now become a global issue that is associated with unintended pregnancies, unwanted births, and unsafe abortions that increase the risk of pregnancy, childbirth-related maternal morbidity, and adverse outcomes for infant and child health [8, 9].

Implanon has been used by more than 4.5 million women worldwide [10]. One of the regions in Ethiopia with an implant utilization rate of 8% was the Southern Nations, Nationalities, and Peoples Regional State [11].

According to data from studies done in Egypt, Kenya, Malawi, Zimbabwe, and Ethiopia, implants are the first option for women; however, the percentage of Implanon users who stop using them early ranges from 17 to 47% [12, 13]. Access to FP services has advanced significantly in Ethiopia. The Federal Ministry of Health (FMoH) of the Ethiopian government pledged to improve the reproductive health (RH) status of Ethiopian women, men, and young people through the four-tiered healthcare delivery system to guarantee the provision of primary health services throughout the nation [13–16]. Additionally, in 1995, the FMoH has collaborated with other partners to enhance RH/FP services at the institution and community levels [17, 18].

Implanon removal has resulted in more than 25 million unwanted births worldwide annually [8, 19]. After discontinuing implanon use for three months, 14 million African women are exposed to the danger of an unwanted pregnancy. For instance, 51% of women are at risk of conception in Egypt, 73% of women in Malawi, 47% of women in Zimbabwe, and 42% of women in Ethiopia removed implanon [13]. Implanon were widely used by sexually active adolescents in Sub-Saharan Africa (SSA), but many of them were stopped before the scheduled appointment, which leads to adverse maternal and child health (MCH) outcomes [11].

The Ethiopian government wants to reduce unmet need to 10% while raising the contraceptive prevalence rate among married women of reproductive age to 55% [15]. However, Ethiopia has a 35% discontinuation rate for modern contraceptive techniques, with an 11% discontinuation rate for implants within a year [11, 20].

The Health Extension Programme (HEP) designed to enhance access to RH care, was introduced by the Ethiopian Ministry of Health in 2003 [21, 22]. In numerous regions of Ethiopia, the rate of Implanon discontinuation continues to be unacceptable [23–26]. Because of its association with negative RH outcomes, high rates of discontinuation of contraceptives for reasons other than decreased necessity pose a public health risk [12, 27].

According to the available information, experiencing adverse effects like unpredictable and irregular vaginal bleeding and not receiving pre-insertion counseling are among the major causes of Implanon discontinuation [7, 28, 29]. The factors influences the discontinuation of implanon are contradicted by many studies. Therefore, this study intends to identify the determinants of Implanon discontinuation among women in Bedelle town public health institutions, Southwest Ethiopia, 2023.

## Methods and materials

### Study setting and design

The institutional-based, unmatched case–control study was conducted in Bedelle town from February 1, 2023, to March 02, 2023. Bedelle Town is the capital city of Buno Bedelle Zone, located in Oromia Regional State at a distance of 408 km south-west of Addis Ababa (the capital city of Ethiopia). The total population was 40,483, of which 13,284 were females in the reproductive age group. In the town, there is 1 health center and 1 public hospital that provide maternal and child health services. The study was done at one health center and one general hospital.

### Source population and study population

#### Cases

All women of reproductive age (15–49 years) who requested Implanon removal in Bedelle town public health institutions before the completion of 3 years.

#### Controls

Included women of reproductive age (15–49 years) who requested Implanon removal in the Bedelle town public health institutions after completion of 3 years.

The study population included all women of reproductive age who requested Implanon removal in the selected public health institutions in the study period.

### Inclusion criteria and exclusion criteria

Women of childbearing age who are eligible for cases and controls and live in the study town were included as study participants. Women of reproductive age who had discontinued the Implanon due to medical complications, failure of the method, or were critically ill at the time of the study period were excluded.

### Operational definition

#### Implanon discontinuation

Implanon discontinuation is the cessation of Implanon use prior to 3 years after insertion.

#### Method failure

Women who get pregnancy while using Implanon as contraceptive.

#### Counseling

Involves making women aware of the long-term protection of the implant, its side effects, its effectiveness, and the advantages of the method.

#### Side effects

Menstrual irregularities, headaches, weight gain, and insertion site pain are examples of side effects, as are the emergence of at least one of these disorders.

#### Sample size determination and technique

The sample size for the study was determined using Epi Info version 7.1 software by considering the following assumptions: a level of significance of 95%, a power of 80%, a ratio of cases to controls of 1:1, a proportion of controls exposed at 31.1%, an odds ratio (OR) of 2.2, and a percent of cases with exposure of 17% from previous similar studies [20]. Thus, the minimum adequate sample size for this study was obtained at 316. By considering a 10% non-response rate, the final sample size turned out to be 348 individuals (i.e., 174 cases and 174 controls). The study was conducted at two public health facilities in Bedelle town, which include Bedelle General Hospital and Bedelle Health Center, and the two were included in the study. The calculated sample size was proportionally allocated to each health facility on the basis of the previous consecutive 6-month average client flow of the units, which was obtained by referring to client registration logbooks. The average 6-month client flow for Implanon removal in the Bedelle General Hospital was 225 cases (325 controls), and in the Bedelle Health Center, 185 cases (267 controls). A total of 410 cases and 592 control women were registered for Implanon removal in each health institution, and, among these, the women who removed Implanon for the purposes of conceiving were excluded from sample allocation. In each selected health facility, the cases were selected consecutively until the required sample size was reached, and the controls were selected using the systematic sampling technique. By using the lottery method to select the first client in each health facility based on the sequence of their family planning visits.

### Data collection method and management procedure

A structured, face-to-face interviewer-administered questionnaire was used to gather the data. It was adapted from the EDHS 2016 and different published works [20, 30–32]. In order to maintain consistency, the questionnaire was first written in English, and then translated into Afan Oromo, and then back into English. A pretest was conducted on 5% of the sample size in the Agaro General Hospital before the actual data collection. Four diploma midwives participated in data collection, with one Bachelor of Science (Bsc) midwife serving as the supervisor. For two days, supervisors and data collectors received training on the study's objectives, the proper timing of data collection, and general data collection techniques.

### Data processing and analysis

The data were checked for completeness, coded, and cleaned before being entered into a computer. Then, it was entered into Epi Data version 4.6 and exported into SPSS version 25.0 for analysis. Data exploration was conducted to assess the completeness, and descriptive statistics like frequencies, tables, and figures were used to describe background variables. A bivariable logistic regression analysis was done for each independent variable, and then, those variables with *p* values ≤ 0.25 were entered into a multivariable logistic regression to control possible confounders. The backward stepwise logistic regression variable selection method with a *P*-value less than 0.05 and an AOR with their respective 95% CI was used to identify independent predictors for Implanon discontinuation. The model's fitness was tested using the Hosmer and Lemeshow goodness of fit test, and the model was declared fit (*P* = 0.240). Finally, the results were presented using tables, graphs, and narration.

## Results

### Socio-demographic characteristics

All sampled 348 women (174 cases and 174 controls) participated in this study, making the response rate 100%. Majority of study participants were protestant, making 97 (55.7%) cases and 89 (51%) for the control group (Table 1).

**Table 1:** Socio demographic and economic characteristics of the respondents in south west Ethiopia, 2023

Variable		Cases(*N* = 174)	Control(*N* = 174)
		no (%)	no (%)
**Women’s age at insertion of implanon(year)**	15–24	47(27)	35(20)
	25–34	84(48.2)	98(51.7)
	25–29	28(16)	30(17.2)
	30–34	12(6.8)	10(5.7)
	+ 35	3(1.7)	1(0.06)
**Religion**	Orthodox	58(33.3)	61(35)
	Muslim	17(9.7%)	19(11)
	Protestant	97(55.7)	89(51)
	Catholic	2(0.11)	5(2.8)
**Can read and write**	Yes	89(51.2)	95(54.5)
	No	85(48.8)	74(42.5)
**Women occupation**	Government employee	66(38)	75(43)
	NGO employee	28(16)	29(16.7)
	Merchant	25(14.3)	27(15.5)
	Housewife	45(25.8)	26(14.9)
	Student	10(5.7)	16(9)
	Daily laborer	0(0)	1(0.006)
**Family Size**	1–3	30(17.2)	31(17.8)
	4–6	124(71.2)	12,571.8)
	7–9	20(11.5)	18(10.3)
**Monthly income**	< 1000	3(1.7)	3(1.7)
	1000–2000	17(9.8)	5(2.8)
	2000–4000	42(24)	22(12.6)
	> 4000	112(63.4)	144(82.7)
**History of abortion**	Yes	56(32)	47(27)
	No	118(67)	127(73)

### Family planning counselling, knowledge, and utilization characteristics

Based on the finding, 174(100%) cases and 174(100%) controls have ever heard about contraceptive methods before inserting implanon. Sources of information about contraceptives for 62(35.6%) cases and 67 (38.5%) controls were health professionals. Additionally, 81(46.5%) of cases and 20(11.5%) of controls were not aware duration of implanon pregnancy prevention (Table 2).

**Table 2:** Contraceptive related characteristics of the respondents in south west Ethiopia 2023

Variable		Cases(*N* = 174)	Control(*N* = 174)
		no (%)	no (%)
Contraceptive information	Ever heard	174(100)	174(100)
Source of information	Friends	48(27.5)	43(24.7)
	HP/HEW	47(27)	43(24.7)
	HC/HW	62(35.6)	67(38.5)
	Media/TV/Radio	17(9.7)	21(12)
Information did you know about contraceptive	Effectiveness	78(44.8)	67(38.5)
	Side effect	33(19)	50(28.7)
	Duration of action	33(18.9)	28(16)
	Benefit	29(16.7)	29(16.7)
Can implanone prevent pregnancy for 3 years	Yes	93(53.5)	142(81.6)
	No	81(46.5)	20(11.5)
Ever used contraceptive before	Yes	133(76.4)	158(91)
	No	40(23.6)	16(9)
Get Counseling service	Yes	174(100)	174(100)
	No	0(0)	0(0)
Type of counselling service	Individual	95(54.59)	64(36.78)
	Mass	62(35.6)	37(21.2)
	With husband	17(9.7)	73(42)
Duration of counseling service	For 15 min	102(58.6)	60(34.4)
	For 15–30 min	62(35.6)	37(21.2)
	More than 30 min	17(9.7)	73(42)
Discuss with partner	Yes	73(42)	151(86.8)
	No	100(57.7)	22(12.6)
Side effect after using implanone	Yes	133(76.4)	62(35.6)
	No	40(23)	111(63.8)
Follow provided	Yes	156(89.6)	165(94.8)
	No	17(9.7)	9(5)
Satisfied by service	Yes	80(46)	134(77)
	No	94(54)	40(23)

### Factors for Implanon discontinuation

**Fig. 1:**
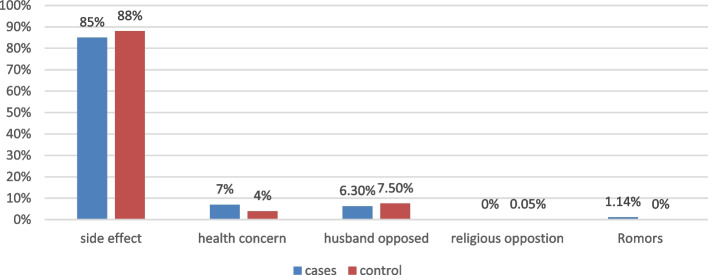
Reason for early removal of implanone among cases and controls in in south west Ethiopia from February 1 to February30, 2023

The most common reasons for early discontinuation in 149 (85%) cases and 153 (88%) controls were side effects. (Fig. 1).

### Types of side effects experienced by respondents

**Fig. 2:**
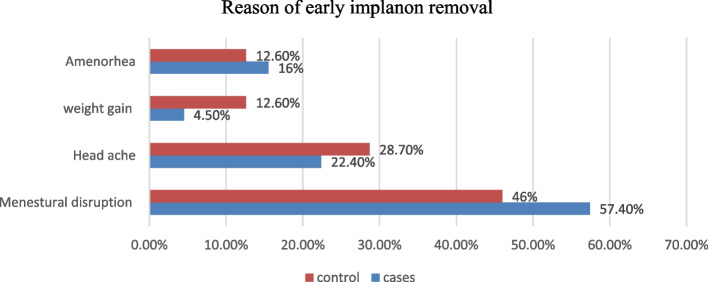
Types Side effects experienced by respondents among cases and controls in in south west Ethiopia from February 1 to February30, 2023

Menstrual disruption was the most common complaint among cases 100 (57.4%) and controls 80 (46%) (Fig. 2).

### Perception of the respondents on Implanon

Of total respondents, about 78 (44.8%) of cases and 88 (50.5%) of control groups perceive that Implanon can cause infertility, and nearly 139 (79.8%) of cases and 73 (42%) believe that implanon can delay menses. majority of cases 97(55.7%) and about 54 (31%) control group believe Implanon has more side effects than other methods. This study also reveals that in about 98 (56%) cases) and 91 (52.3%) controls, Implanon better prevents pregnancy than other methods, despite its side effects.

### Determinant of early Implanon discontinuation

Husband's educational status; implanon is immediately reversible; previous history of contraceptive use; type of counseling they obtained; time spent on counseling; discussion with their partner; Side effect after Implanon, change in the amount of menstrual flow, satisfaction with the service given, type of side effect, perception that Implanon delayed menses, and thinking Implanon had more side effects than others were variables found to be statistically significant in a bivariate model at a p-value less than 0.25 and after adjudicating with multivariable logistic regression, husbands educational status, married women who obtain individual and mass counseling, women who counseled less than 15 min during Implanon insertion, women who discuss with their partners before insertion, side effects after Implanon use, and women who were satisfied with the service given were found to be statistically significant predictors for Implanon discontinuation in multivariable analysis (Table 3).

**Table 3:** Factors associated with discontinuation of implanone among married women who discontinue implanone in south west Ethiopia 2023

Variable		Cases	Control	COR (95%CI)	AOR (95%CI)	*P*-value
**Husband education status**	Yes	142	160	2.495(1.276–4.877)^*^	.338(0.121–0.944) **	.038
	No	32	14	1	1	
**Implanone immediately reversible**	Yes	94	137	3.151(1.970–5.041)^*^	.526(.249–1.107)	.091
	No	80	37	1	1	-
**History previous contraceptive use**	Yes	133	158	2.970(1.591–5.543)^*^	0.962(0.369–2.509)	.937
	No	40	16	1	1	
**What type of counseling did you obtain**	Individual counseling	95	64	0.157(0.085–0.290)*	3.403(1.390–8.332)^**^	.007
	Mass counseling	62	37	0.139(0.071–0.271)^*^	5.687(2.151–15.033)**	.000
	With husband counseling	17	73	1	1	
**Time spent for counseling**	For 15 min	102	60	0.204(0.118–0.353)*	3.243(1.303–8.046)**	.011
	For 15–30 min	46	39	0.294(0.159–0.545)^*^	2.036(0.803–5.163)	.134
	More than 30 min	26	75	1	1	
**Discuss with your partner**	Yes	73	151	0.106(0.062–0.182)^*^	0.289(0.143–0.585)^**^	.001
	No	100	22	1	1	
**Side effect after implanone**	Yes	133	62	0.168(0.105–0.269)^*^	3.989(2.069–7.690)^**^	.001
	No	40	111	1	1	
**Satisfied by service given**	Yes	80	134	3.877(2.441–6.158)^*^	.371(0.166–0.828)^**^	.004
	No	94	40	1	1	
**Type of side effect**	Menstrual disruption	100	80	0.291(0.123–0.668)^*^	2.430(.662–8.916)	.181
	Amenorrhea	27	22	0.296(0.111–0.794)^*^	1.679(.383–7.535)	.492
	Head ach	39	50	0.466(0.187–1.159)	1.536(0.382–6.183)	.546
	Weight gain	8	22	1	1	
**Perceive Implanone delay menses**	Yes	139	73	0.182(0.113–0.293)^*^	2.943(0.381–6.274)	.045
	No	35	101	1	1	
**think implanone more side effect than others**	Yes	97	54	0.357(0.230–0.554)^*^	1.400(.703–2.786)	.339
	No	77	120	1	1	

*Key note: 1= reference category : ** *P*-value significant <0.05

## Discussion

In this study, women whose husbands have a formal education had 77 percent delayed chances of discontinuing implanon earlier than those whose husbands have no formal education. This finding was comparable with a study conducted in northern Ethiopia [27, 33]. The possible reason could be in our current study eight in ten husbands have formal education, which might increase their understanding of the duration of implanon removal time.

The odds of early removal were three times higher among those advised individually than those counselled with their partners. These were in line with a study conducted in Debra Berhan, Ethiopia [32]. The possible consistency could be methodological similarity as well as the background characteristics of the study population. In addition, our study found that women who received counselling for less than five minutes were three times more likely to discontinue using an implanon than women who received counselling for more than thirty minutes. This is supported by studies conducted in the Tigray region and Ambo [25, 34]. The consistency with which long-term counselling enhances understanding and acceptance of health care counselling is one possibility.

Furthermore, those who communicated with their partner before insertion are 71% less likely than their counterparts to get rid of their implanon early. It is comparable to the study conducted in Bahirdar, Ethiopia [35]. It could be because couples' mutual interest in having children raises the possibility that they will cease using, as well as possible methodological similarity in the study.

Implanon was discontinued six times more frequently in women who experienced side effects than in women who did not experience side effects. The results were consistent with those of research conducted in Gamo Gofa, Mekelle, and Bahir Dar, Ethiopia [35–37]. The plausible reason could be given that side effects interrupt and exacerbate inconsistent early management implanon side effects, as well as study participants' similarity.

In our findings, 85.2% of women who were satisfied with the service during implantation were less likely to discontinue early than women who were not satisfied with the service. This finding is agreed with the study conducted in Tigray region, Ethiopia [4, 25, 38]. It might be related with implanon cessation would be delayed for those who received satisfactory service help to explain the reasoning behind this choice.

### Limitation of study

Women may have difficulty remembering when she inserts implanon, which means recall bias. Since, the study was conducted in urban health facility setting, though most of the population lives in rural area.

## Conclusion

Implanon discontinuation was predicted by the husband's education; the number of women counselled alone, the length of counselling, a discussion with the spouse, satisfaction with the service, and implanon side effects. The health care provider should increase counselling services, especially length of implanon pregnancy prevention, in accordance with the national family planning recommendations, to reduce early implanon removal.

## Data Availability

Data we used in manuscript available from the corresponding author upon reasonable request.

## Abbreviations

AOR: Adjusted Odd Ratio
EDHS: Ethiopian Demographic Health Survey
FMoH: Federal Ministry of Health
HEP: Health Extension Program
WHO: World Health Organization
